# The Book World of Medicine and Science

**Published:** 1905-03-18

**Authors:** 


### The Book World of Medicine and Science.

The Diagnosis and Modern Treatment of Pulmonary Consumption. By Arthur Latham, M.D., F.R.C.P. Second Edition. (London: Baillière, Tindall and Cox. 1905. Pp. 224. Price 5s. net.)

Dr. Latham's handbook has already reached a second edition and has been revised without marked change in its essential features. It still remains one of the most reliable manuals on the open-air treatment of phthisis as practised in the best sanatoria or as adapted to the requirements of a single patient in a private house. Though not to be regarded as substitutes for the open-air system, the author deals in some detail with other modern methods of treatment such as tuberculin, antiseptic injections and inhalations, cantharidate of potash, and Marmorek's serum, with remarks on the use of such remedies as Friar's balsam, creasote, and urea. A chapter on the treatment of special symptoms contains some excellent advice on the uses and limitations of cough mixtures in phthisis and the great importance of a careful and exhaustive search for the cause of the cough which is often but indirectly connected with the patient's pulmonary condition. The author's remarks on the relation of marriage to tuberculosis deserve attention as somewhat more favourable than those usually expressed, but the statistics of Squire as to the incidence of tubercle in the children of phthisical parents seem to justify more hopeful views on this question.

The Open-Air Treatment of Pulmonary Tuberculosis. By F. W. Burton-Fanning, M.D. (London: Cassell and Co. 1905. Pp. 176. Illustrated. Price 5s.)

This little volume forms a handy guide to the principles and practice of open-air treatment in phthisis. It is characterised throughout by detail, the attention to which is so essential in such cases. While admitting that the benefits of this method of treatment are not confined to the less advanced cases of pulmonary affection, the author rightly insists on the importance of the early diagnosis of phthisis if the best results are to be obtained, and in this connection his remarks on the character of the temperature chart in suspected cases are of great importance. The chapter on diet in consumption is also of special value as a plain statement of what is aimed at in the feeding of the subjects of pulmonary tuberculosis. It should do much to dispel the vague statements as to the part played by "over-feeding" in sanatorium treatment which have to some extent found credence with the public to the detriment of these institutions. The author directs attention to the care required by patients after leaving the sanatorium and appeals to the public for co-operation in finding light employment for such persons in suitable surroundings. Some useful information is appended as to the most suitable forms of shelters and other appliances for use in carrying out the treatment at the patient's home.

A Dictionary of New Medical Terms. By George M. Gould, M.D. (London: Baillière, Tindall and Cox. 1905. Pp. 571. Price 21s.)

The phenomenal growth of medical science during recent years has inevitably resulted in the creation of a great number of new technical expressions and names, but the extent to which this process of creation has reached at the present day is probably not generally appreciated. Dr. Gould in his preface says that no fewer than 30,000 new terms have passed into currency during the last ten years. This growth has led him to prepare the present work as a supplement to his "Illustrated Dictionary of Medicine," which appeared a decade ago. Very wisely, we think, the author has refrained from differentiating between the names that are likely to be retained in general use and those that are almost certain to become obsolete; all new medical terms, good and bad alike, are interpreted. Although the volume includes definitions of no fewer than 38,000 words, it has been found possible, by an ingenious arrangement of abbreviations, to preserve for the book a size which is compatible with facile handling and reference—by no means a small advantage to the user. The dictionary fills a much-felt want, and certainly will prove of great value to all medical men who wish to keep themselves up to date.

The Radical Cure of Corns and Bunions. By E. Harding Freeland, F.R.C.S. (London: John Bale Sons and Danielsson, Limited. Pp. 34. Price 1s. Second edition.)

Mr. Freeland's brochure contains some facts about corns and bunions which ought to prove useful, considering the frequency of these conditions. He advocates excision of the corn, and also of the bursa found beneath it, and quotes successful cases. The illustrations are good, and add to the value of the book.

Religion and Health: Their Mutual Relationship and Influence. By Norman Porritt, M.R.C.S., L.R.C.P. (London: Skeffington and Son. 1905. Price 3s. 6d.)

Mr. Norman Porritt's book does more than detail the analogies between religion and health, though he gives much curious information as to the relation which has existed between the two from their earliest developments until now. He draws an instructive lesson from the advantages enjoyed by those whose attention to the laws of health is all the stronger that it has a religious basis. To nothing else than their scrupulous adherence to the Mosaic law, with its minute rules for eating, fasting, and cleansing can be attributed the superior health and longevity which Jews enjoy over Gentiles, a superiority which is most manifest among those who live in the worst environments. Similarly abstainers and those who are most abstemious in eating and drinking have the best records in the health tables of the insurance companies. Ably written, with a wealth of novel and interesting information, Mr. Porritt's book should be valuable to clergymen and doctors, and to the public at large also.

The Transvaal Burgher Camps, South Africa. By Lieut.-Col. Thomson, C.I.E., I.M.S., late Director of Burgher Camps, Transvaal. (Allahabad: The Pioneer Press. Pp. 74. Price 2 rupees.)

Col. Thomson has written a very readable little book, giving the general plan of the camps in the Transvaal and details of their management. The tables of figures show, in a striking manner, the great decrease of mortality after more efficient methods of sanitation and hygiene had been adopted. The book, though small, contains a great deal of information, and is well worth the notice of sanitarians.

---

In the review of Keith's "Human Embryology and Morphology," which appeared in our columns on the 4th inst., the price of the book was incorrectly stated to be 21s. 6d. The correct price is 12s. 6d.

